# Investigation of the Enhancement Interactions between Double Parallel Cracks on Fatigue Growth Behaviors

**DOI:** 10.3390/ma13132952

**Published:** 2020-07-01

**Authors:** Zhichao Han, Caifu Qian, Huifang Li

**Affiliations:** Institute of Mechanical and Electrical Engineering, Beijing University of Chemical Technology, Beijing 100029, China; hanzhichaohzc@163.com (Z.H.); qiancf@mail.buct.edu.cn (C.Q.)

**Keywords:** crack interaction, double parallel cracks, crack growth rate, stress intensity factor

## Abstract

In this paper, interactions of double parallel cracks were studied by performing experiments and numerical simulations. Fatigue crack propagation tests were carried out to measure crack growth rates in the specimens with double parallel cracks or a single crack. Finite element method was adopted to calculate stress intensity factors at the crack tips. Results show that the double parallel cracks at different positions present a shielding effect or enhancement effect on crack growth rates and stress intensity factors. When the double parallel cracks are offset, crack interactions mostly behave as enhancement effects. Empirical formulas were obtained to calculate the stress intensity factor at the “dangerous” crack tip of the double parallel cracks. By modifying the material parameters in Paris equation of the single crack, the double parallel cracks are simplified into a single crack with the same crack growth rates.

## 1. Introduction

Multiple cracks usually initiate in the weld joints, because the weld butt and fillet contain heterogeneous microstructures, leading to high residual stresses at weld toes [1,2,3]. Due to the particles and voids in the coatings, cracks may appear in these locations [4]. Additionally, multiple cracks can easily generate at the structures, such as rivet holes, which are usually affected by the stress concentration [5]. Then, multiple cracks propagate easily under the fatigue loadings [6]. In the process of initiation and growth, multiple cracks may affect or be affected by the adjacent cracks, i.e., multiple cracks experience interactions [7]. During the fatigue life estimation, multiple cracks in the components are usually equivalent to a single crack, but crack interactions on the stress intensity factor (SIF) and crack growth rate (CGR) are usually assessed qualitatively, leading to the simplification not so accurate [8,9,10]. So, it is significant to investigate the interactions of multiple cracks in terms of CGRs.

Crack interactions would change the stress field around the crack tips [11], leading to the values of the stress decreasing or increasing; therefore, the SIFs at crack tips receive a shielding or an enhancement effect [12,13,14]. Chai et al. [15] analyzed interaction between an embedded crack and surface crack in a cylindrical pressure vessel. They found that the crack interaction increased the SIFs of the embedded crack and the surface one in comparison with the single embedded crack and the single surface one. Kim et al. [16] found that failure probability of the structures decreased with the number of cracks increasing because the shielding effect reduced the stress field around all the cracks. Zhao et al. [17] and Li et al. [18] researched interactions of multiple offset parallel cracks, and results showed that crack interactions could afford either a shielding or enhancement effect on the SIFs and stress field determined by the relative positions and sizes of cracks. Jiang et al. [19,20] found that due to the interactions of double parallel cracks, the tensile stress around the crack tips was released and SIFs at crack tips decreased.

Besides stress intensity factors, crack interactions also affect the growth behaviors of multiple cracks, such as crack growth paths and crack growth rates [21,22]. Kishida et al. [23] researched the growth behaviors of three parallel cracks. Results showed that because multiple cracks experienced interactions, the longest crack might not receive the greatest tangential stress or the SIF at crack tip. Sun et al. [24] and Jin et al. [25] found that the double parallel cracks propagated towards each other in the crack growth process because stress redistributions around the crack tip changed the location of the maximum circumferential stress. Wang et al. [26] conducted experiments on multiple-site damage crack propagation behaviors. Results showed that as cracks grew towards each other, the crack growth accelerated. Gope et al. [27] found that for the double offset cracks, propagation paths of the inner crack tips exhibited a mutual attraction determined by the positions and sizes of the crack tips, whereas the outer crack tips still propagated perpendicularly to the direction of load. Jin et al. [28] found that the double collinear cracks and the single crack had different material parameters of Paris equation. Therefore, a new driving force, Δ*K*_n_, was introduced, which was obtained from the net section stress range Δ*σ*_n_. Thus, based on the new driving force, Δ*K*_n_, Paris equations of the single crack and double collinear cracks showed little difference. Kamaya et al. [29,30] investigated the multiple interacting surface cracks with numerical simulation and fatigue tests. Results showed that for the same Δ*K*_I_, the double parallel surface cracks had lower CGRs than the single surface crack. Thus, the crack growth area *A* is considered to be the representative parameter in crack propagation. Results showed that the new crack growth rate, d*A*/d*N*, showed good correlation with the new driving force, *σA*^0.5^, between the single surface crack and double parallel surface cracks.

In this paper, interactions of double parallel cracks were studied by performing experiments and numerical simulations. Crack growth rates of the double parallel crack and single crack specimens were obtained through the fatigue crack propagation tests. Finite element method (FEM) was adopted to calculate the SIFs at the double parallel crack tips. Experiments and simulation results showed that the CGRs and SIFs at crack tips received a shielding or enhancement effect. Empirical formulas were obtained to calculate the SIF at the “dangerous” tip of the double parallel cracks. By modifying the material parameters in Paris equation for the single crack, the double parallel cracks are simplified into a single crack with the same CGRs.

## 2. Experiment Measurement

### 2.1. Specimen Preparation

The specimens are machined from the hot-rolled plate of S30408 stainless steel with the dimensions of 260 mm × 48 mm × 6 mm. The chemical composition (wt%) and the mechanical properties of the steel are listed in Table 1 and Table 2.

**Figure 1:**
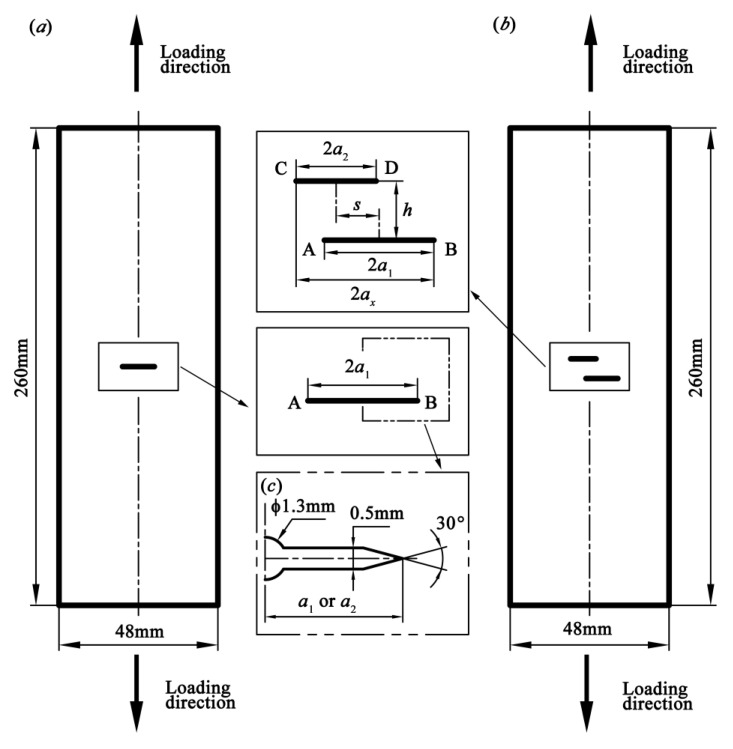
Specimen geometry: (**a**) specimen with single crack, (**b**) specimen with double parallel cracks, and (**c**) dimensions of the notch configuration (initial crack).

Figure 1a illustrates the geometry of the specimen containing a single crack of length 2*a*_1_. Crack tips are denoted by letters A and B. Figure 1b depicts the geometries of the specimens with double parallel cracks, which are defined as crack 1 and crack 2 with the lengths 2*a*_1_ and 2*a*_2_, respectively, where *a*_1_ ≥ *a*_2._ The normal and offset distances of the double cracks are defined as *h* and *s*, respectively. Crack tips of the double parallel cracks are denoted by letters A–D. The loading direction is perpendicular to the cracks, as shown in Figure 1. Table 3 shows the initial sizes and positions of the cracks in seven groups of specimens, where SC denotes the single crack, PC1.0s4 denotes the double parallel cracks for *a*_2_/*a*_1_ = 1.0 and *s* = 4 mm, PC0.7s6 denotes double parallel cracks for *a*_2_/*a*_1_ = 0.7 and *s* = 6 mm, and so on. In addition, for the double parallel cracks, the projected length of the initial double parallel cracks, i.e., the one perpendicular to the loading direction, 2*a_x_*, can be calculated by Equation (1). Figure 1c shows the dimensions of the notch configuration (initial crack).

(1) $$2a_{x}=a_{1}+a_{2}+s$$

In this study, the bisector line of the initial projected crack coincides with the centerline of the plate.

### 2.2. Fatigue Test Settings

Fatigue crack propagation tests were performed on an INSTRON 8800 fatigue testing machine (Instron Engineering Corporation, Boston, MA, USA) at the room temperature under laboratory air conditions. Constant stress ratio, maximum load, and frequency of the sinusoidal loading wave form were 0.1, 40 kN, and 45 Hz, respectively. The fatigue crack growth paths and lengths were recorded with the digital microscope system (Mshot MS60, version 1.3.11, Mingmei Optoelectronic Technology Corporation, Guangzhou, China).

### 2.3. Tests Results

#### 2.3.1. Crack Growth Paths

**Figure 2:**
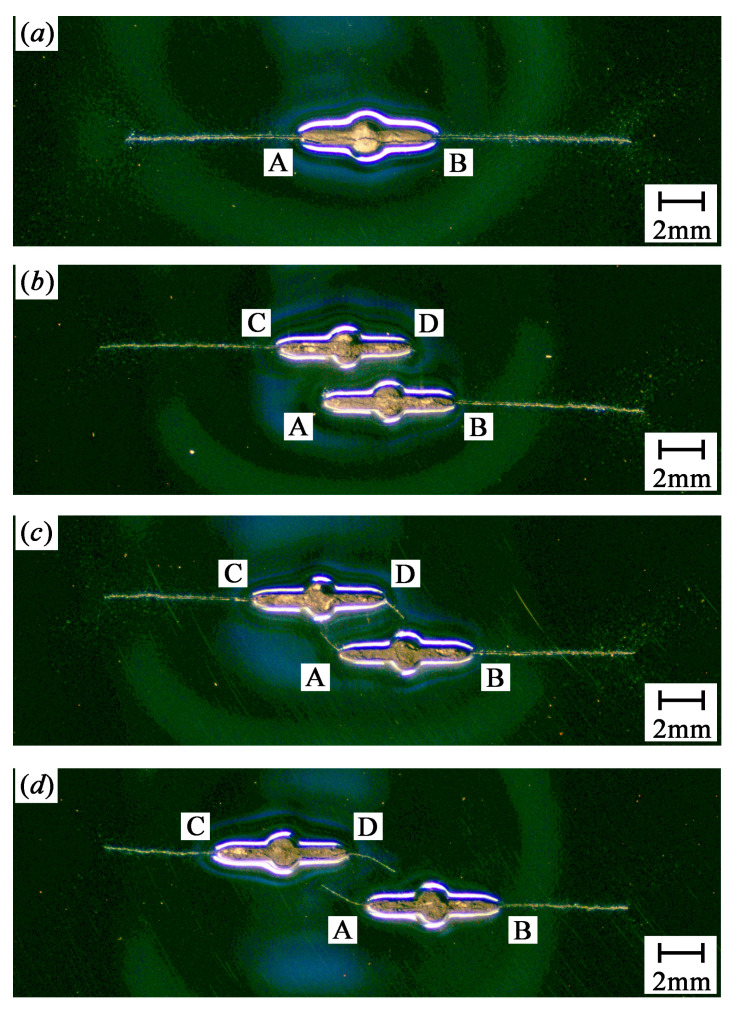
Crack growth paths for different specimens: (**a**) SC, (**b**) PC1.0s2, (**c**) PC1.0s4, and (**d**) PC1.0s7.

**Figure 3:**
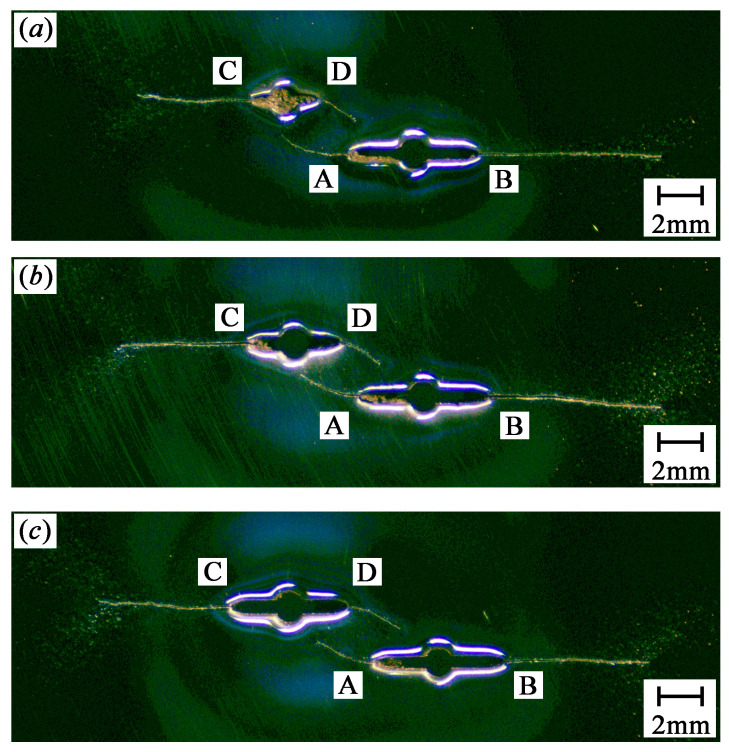
Crack growth paths for different specimens: (**a**) PC0.5s6, (**b**) PC0.7s6, and (**c**) PC0.9s7.

Figure 2 shows the crack growth paths for SC, PC1.0s2, PC1.0s4, and PC1.0s7 specimens. Figure 3 shows the crack growth paths for PC0.5s6, PC0.7s6, and PC0.9s7 specimens.

As shown in Figure 2 and Figure 3, the two crack tips of the single crack specimen propagate perpendicularly to the loading direction. Similarly, the outer crack tips (crack tips B and C) of the double parallel crack specimens propagate perpendicularly to the loading direction until the specimens fracture, whereas the inner crack tips (crack tips A and D) propagate toward the adjacent crack because of the crack interaction. In addition, the inner crack tips propagate at low rates than the outer crack tips. For the double equal crack specimens (PC1.0s2, PC1.0s4, and PC1.0s7), the more the initial cracks overlapped, the smaller length the inner crack tips propagate.

#### 2.3.2. Crack Growth Rates

**Figure 4:**
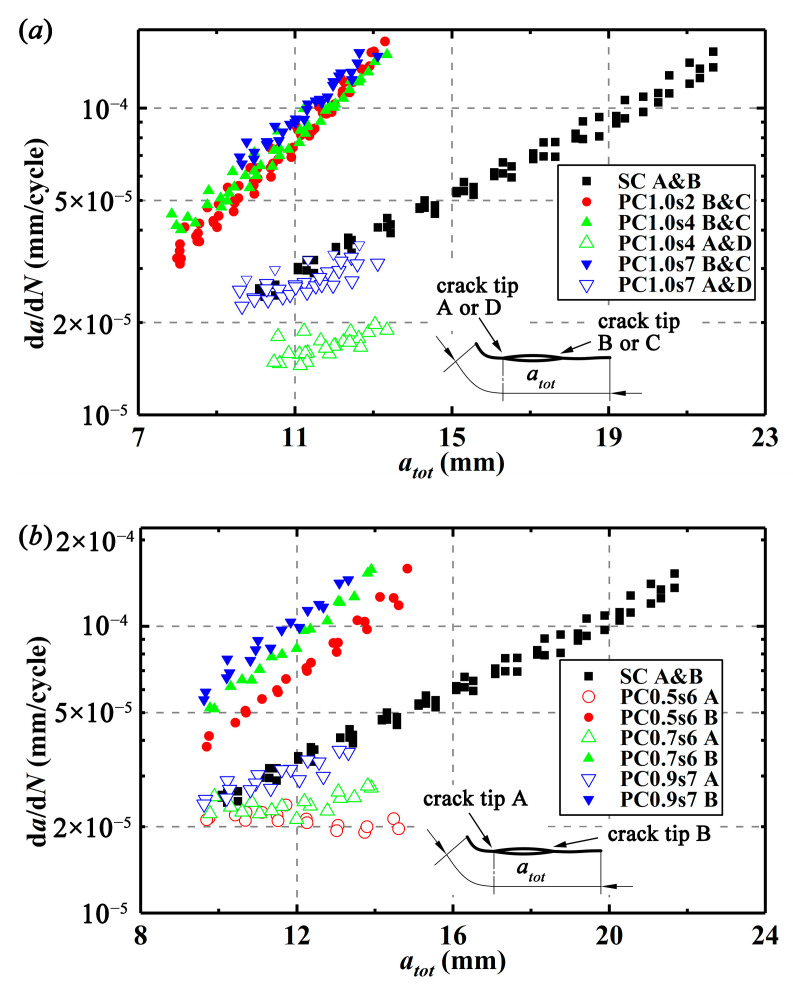
Crack growth rates vs total crack growth length: (**a**) for double equal cracks and single crack and (**b**) for double unequal cracks and single crack.

For comparison of crack interactions, relationships between total crack growth lengths, *a_tot_*, in the crack growth process and crack growth rates, d*a*/d*N*, for different specimens are drawn in Figure 4. The crack growth rates can be calculated by Equations (2)–(5), where *ậ_i_* is the *i*’th crack growth length which is calculated from the center of the initial crack (notch), *b*_0_, *b*_1_, and *b*_2_ are regression parameters obtained by the least-square method, and *N_i_* is the number of cycles corresponding to *ậ_i_*. As shown in Figure 4a, the inner crack tips in PC1.0s2 specimens do not propagate, and in PC1.0s4 specimens, the inner crack tips present much smaller CGRs than the SC specimens, implying that the inner crack tips in these two kinds of specimens experience a great shielding effect. For the PC1.0s7 specimens, with crack propagation, the CGRs at the inner crack tips are getting smaller than those of the SC specimens, implying that the shielding effect increases. For all the double equal crack specimens, the outer crack tips show larger CGRs than the SC specimens. With crack propagation, the difference of CGRs between the single crack specimens and the outer crack tips in the double equal crack specimens is getting larger and larger, indicating that the outer crack tips experience an increasing enhancement effect. Moreover, the larger the initial projected crack length in the double equal crack specimens and the greater the enhancement effect on the outer crack tips, the smaller the shielding effect on the inner crack tips during the crack propagation.

(2) $${\hat{a}}_{i}=b_{0}+b_{1}\left(\frac{N_{i}-C_{1}}{C_{2}}\right)+b_{2}{\left(\frac{N_{i}-C_{1}}{C_{2}}\right)}^{2}$$

(3) $${\left(\frac{da}{dN}\right)}_{{\hat{a}}_{i}}=\frac{b_{1}}{C_{2}}+\frac{2b_{2}\left(N_{i}-C_{1}\right)}{C_{2}^{2}}$$

(4) $$C_{1}=\frac{1}{2}\left(N_{i-3}+N_{i+3}\right)$$

(5) $$C_{2}=\frac{1}{2}\left(N_{i+3}-N_{i-3}\right)$$

As drawn in Figure 4b, for the double unequal crack specimens (PC0.5s6, PC0.7s6, and PC0.9s7), the CGRs at the two crack tips of crack 1 show the same changing trend as those of PC1.0s7 specimens.

**Figure 5:**
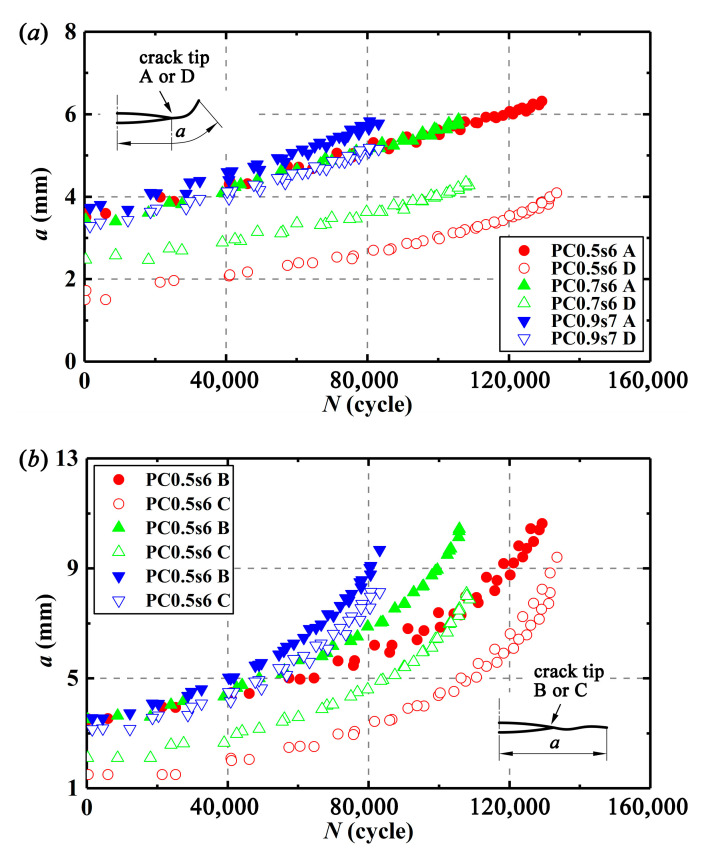
Crack growth length vs. cycle number: (**a**) inner crack tips of the double unequal cracks and (**b**) outer crack tips of the double unequal cracks.

For the double unequal crack specimens, crack growth length, *a*, which is calculated from the center of the initial crack (notch), and number of cycles, *N*, starting at the cycle when crack tip B propagate to 3.5 mm, are drawn in Figure 5. It can be seen that at a given number of cycles, crack tips A and B have larger crack growth lengths compared with crack tips D and C, respectively, or in other words, the crack growth lengths of crack 1 (the long crack) are larger than those of crack 2 (the short crack), indicating that crack 1 is more “dangerous”.

## 3. Numerical Simulations

### 3.1. Finite Element Modeling

**Figure 6:**
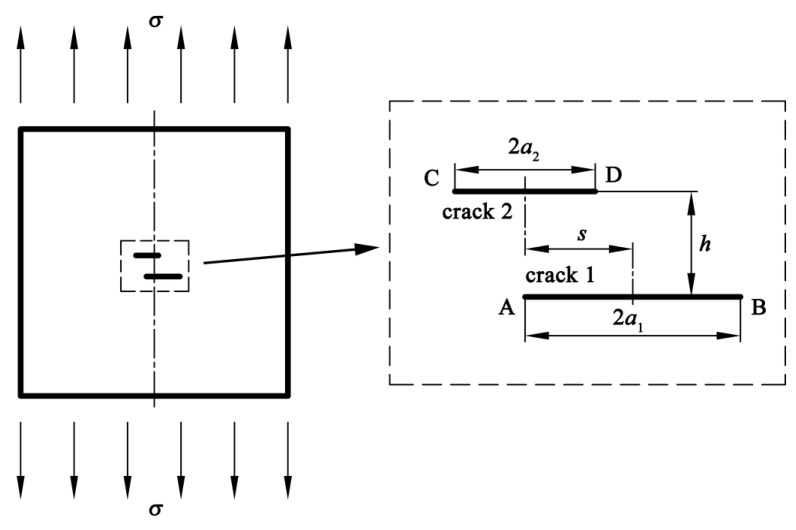
Finite element model of a plate with double parallel cracks.

Crack interactions are usually evaluated with the SIFs at crack tips. Therefore, finite element models of a plate containing double parallel cracks under the uniform remote tension stress *σ* of 125 MPa are established to calculated the SIFs with the linear-elastic analysis, as shown in Figure 6. The plate is considered to be infinite as the crack sizes are much smaller than the plate.

**Figure 7:**
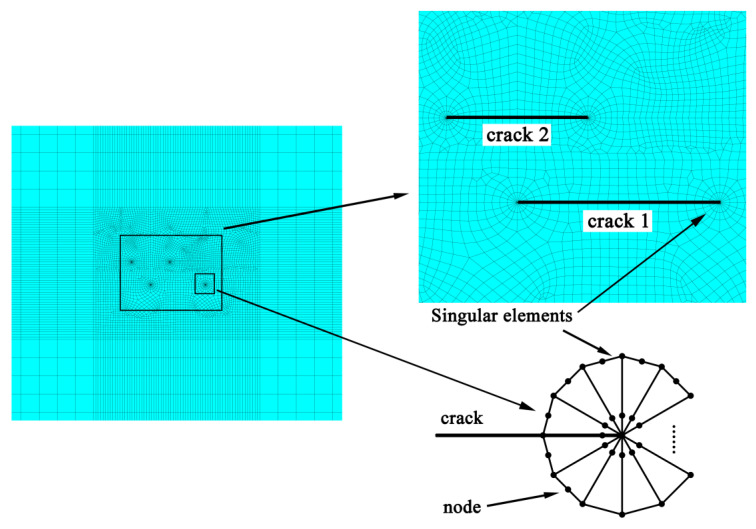
Mesh model of the plate with double cracks.

Mesh generation of the finite element model is completed with high order quadrilateral plane elements. As shown in Figure 7, for the improvement of the calculation accuracy, meshes around the crack tips are refined. Particularly, singular elements are generated at the crack tips to compute SIFs of the double parallel cracks.

### 3.2. Simulation Results of the SIFs

It can be found from Section 2.3.2 that crack 1 is more “dangerous” than crack 2. Considering that for the double parallel cracks, Mode II SIF, *K*_II_ is much smaller than Mode I SIF, *K*_I_ for a given *s* and *h* [11], therefore, Mode I SIF, *K*_I_, at crack tips A or B in crack 1 is calculated. In addition, the SIF of a single crack in an infinite plate with length of 2*a*_1_ is also calculated by Equation (6) for comparison.

(6) $$K_{I}^{0}=\sigma \sqrt{\pi a_{1}}$$

The ratio of the SIF of crack 1 to the SIF of the single crack, *K*_I_/*K*_I_^0^, is defined to represent interactions of the double parallel cracks. Particularly, if the value of *K*_I_/*K*_I_^0^ is less than 1, it can be considered that the crack tip receives a shielding effect on the SIF from the adjacent crack, and if *K*_I_/*K*_I_^0^ is greater than 1, the crack tip receives an enhancement effect on the SIF. Crack interactions can be neglected if *K*_I_/*K*_I_^0^ is close to 1.

The value of *K*_I_/*K*_I_^0^ can be calculated from the geometrical parameters, *h*, *s*, *a*_1_, and *a*_2_. Therefore, three dimensionless parameters, *H*, *S*, and *Ra,* are defined as follows to find the expressions of *K*_I_/*K*_I_^0^.

(7) $$H=\frac{h}{a_{1}}$$

(8) $$S=\frac{s}{a_{1}}$$

(9) $$Ra=\frac{a_{2}}{a_{1}}$$

**Figure 8:**
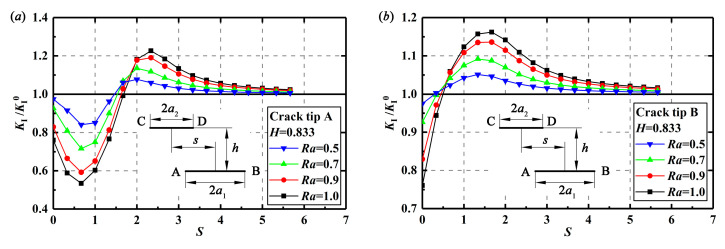
Relationship between *K*_I_/*K*_I_^0^ and *S* with different *Ra* at *H* = 0.833: (**a**) *K*_I_/*K*_I_^0^ at crack tip A and (**b**) *K*_I_/*K*_I_^0^ at crack tip B.

Figure 8 depicts relationships between the dimensionless offset distance *S* and the value of *K*_I_/*K*_I_^0^ in crack 1 at *H* = 0.833 with different *Ra*. It is found that crack 2 has different effects on crack tips A and B. When the value of *S* is equal to 0, crack 2 presents a shielding effect on the SIFs at the two crack tips of crack 1. With the increasing *S*, the shielding effect on the SIFs at crack tip A increases but decreases at crack tip B. When *K*_I_/*K*_I_^0^ at crack tip A reaches the minimum value or the SIFs at crack tip A experience the greatest shielding effect, the SIFs at crack tip B start to change from the shielding effect to the enhancement effect. As the value of *S* continues to increase, the shielding effect on the SIFs at crack tip A decreases, while the enhancement effect at crack tip B increases. When *K*_I_/*K*_I_^0^ at crack tip B reaches the maximum value or the SIFs at crack tip B experiences the greatest enhancement effect, the SIFs at crack tip A change from the shielding effect to the enhancement effect. With further increasing *S*, the enhancement effect on the SIFs at crack tip A increases, while decreases at crack tip B. When double cracks are not overlapped and crack tips A and D are close, SIFs at crack tip A receive the greatest enhancement effect. As the value of *S* is large enough, or in other words, the two cracks are offset enough, the crack interaction vanishes.

**Figure 9:**
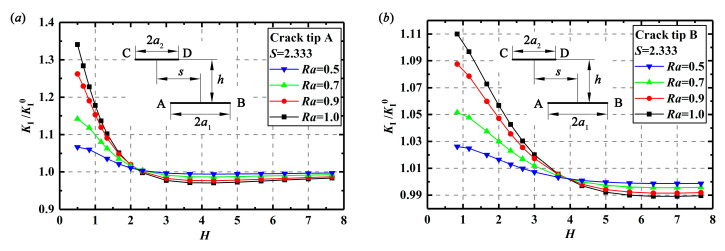
Relationship between *K*_I_/*K*_I_^0^ and *H* with different *Ra* at *S* = 2.333: (**a**) *K*_I_/*K*_I_^0^ at crack tip A and (**b**) *K*_I_/*K*_I_^0^ at crack tip B.

Changes of *K*_I_/*K*_I_^0^ at crack tips A or B with the increasing *H* at *S* = 2.333 are drawn in Figure 9. It seems that with the increase of *H*, the enhancement effect on SIFs at crack tips A or B decreases, and finally, vanishes until the distance of the double cracks is large enough. It is also observed from Figure 8 and Figure 9 that the parameter *Ra* also has influence on the interactions of the double parallel cracks. Actually, a larger *Ra* or a close length of the double cracks will produce a greater shielding or enhancement effect with the change of *S* and *H*.

As the enhancement effect is more “dangerous” compared with the shielding effect in the crack propagation process, more attention should be paid on the enhancement effect at the “dangerous” crack tip, or crack tip B, in the following analysis. Corresponding to the value of *K*_I_/*K*_I_^0^ at crack tip B in Figure 8b changing from the shielding effect to the enhancement effect, the dimensionless offset distance is denoted as *S*_1_, which can be obtained by Equations (10) and (12). Corresponding to the maximum values of *K*_I_/*K*_I_^0^ at crack tip B in Figure 8b for the greatest enhancement effect, the dimensionless offset distance is denoted as *S*_2_, which can be expressed by Equations (11) and (13).

for *H* ≤ 2
  (10) $$S_{1}=-0.163+0.240Ra^{1.770}+0.485H^{1.105}$$
  (11) $$S_{2}=2.315+Ra^{-0.478}\left(-0.665-0.360H+0.348H^{2}\right)$$
for *H* > 2
  (12) $$S_{1}=0.588H-0.123$$
  (13) $$S_{2}=1.107H+0.034$$

With the sufficient numerical results, *K*_I_/*K*_I_^0^ at crack tip B can be expressed with *H*, *S*, and *Ra* as follows:

for *S*_1_ ≤ *S* ≤ *S*_2_
  (14) $$\frac{K_{I}}{K_{I}^{0}}=0.993+0.265Ra^{1.114}e^{-1.072H}S$$
for *S* > *S*_2_
  (15) $$\frac{K_{I}}{K_{I}^{0}}=1.005+0.587Ra^{2.185}H^{-0.300}S^{-2.420}$$

Equations (14) and (15) are valid for 0.5 ≤ *Ra* ≤ 1, *H* ≥ 0.1 and *S* ≥ *S*_1_. With *K*_I_/*K*_I_^0^ obtained from Equations (14) and (15) and by applying Equation (6), the SIF at crack tip B can be easily calculated with the enhancement effects of the adjacent crack. Table 4 lists the comparison results of the SIFs calculated by Equations (14) and (15) or by FEM. It can be found that the relative errors are less than 5.0%, which is acceptable in engineering.

### 3.3. Mechanical Explanation of the Crack Interactions

**Figure 10:**
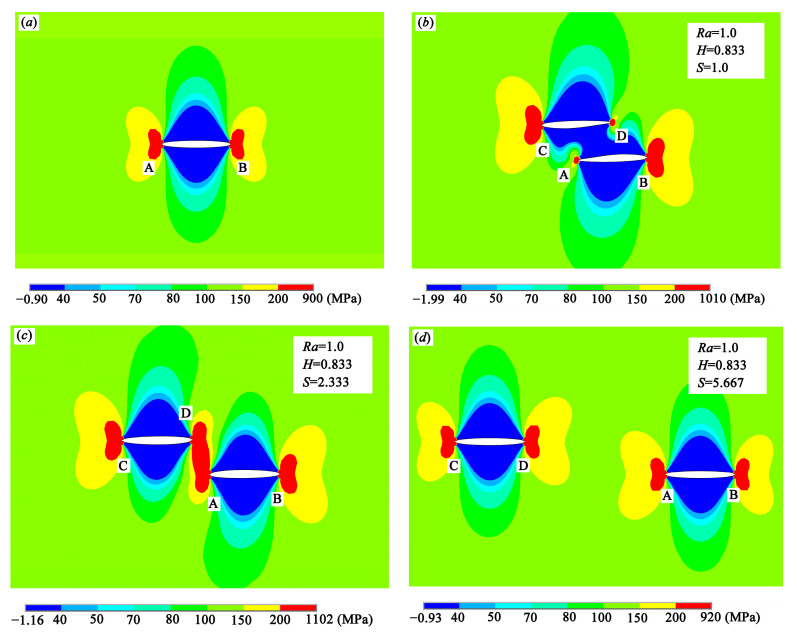
Tensile stress distributions around the crack tips: (**a**) single crack, (**b**) double cracks with *S* = 1.0, (**c**) double cracks with *S* = 2.333, and (**d**) double cracks with *S* = 5.667.

It is found from Figure 8 that crack tips A and B experience the shielding effect or enhancement effect depending on different offset distance. Considering that *K*_II_ is much smaller than *K*_I_, implying that the shear stress is much smaller than the tensile stress, therefore, the tensile stress distributions around the crack tips along the loading direction are investigated to find out the mechanism of the crack interactions from mechanical point of view. Figure 10 shows the tensile stress distributions of the single crack and the double equal cracks with *S* = 1.0, *S* = 2.333, and *S* = 5.667. As shown in Figure 10b, because of the shielding effect, the tensile stress around crack tip A decreases, leading to the decrease of the SIF at crack tip A. However, the tensile stress around crack tip B is enhanced and thus, SIF at this crack tip is raised as the effective length of two cracks increases. However, it is found from Figure 10c that when *S* is increased to some value (see *S* > 2 in Figure 8a), the tensile stress around the crack tips A and D is also enhanced, and the SIFs at these crack tips increase. In addition, it is found from Figure 10d that if the double cracks are far away, crack interactions fade away.

## 4. Crack Growth Rates of Double Cracks

It is found in Figure 2 and Figure 3 that for the double parallel crack specimens listed in Table 3, the growth lengths of the inner crack tips are relatively small. The outer crack tips propagate until the specimens fracture. Therefore, for the double parallel crack specimens, more attention should be paid on the crack propagation at the outer crack tips.

In crack growth process, the relationship between the driving force, Δ*K*_I_, and crack growth rate, d*a*/d*N*, is usually described by the Paris equation [34], as seen in Equation (16).

(16) $$\frac{da}{dN}=c{\left(\Delta K_{I}\right)}^{m}$$

**Figure 11:**
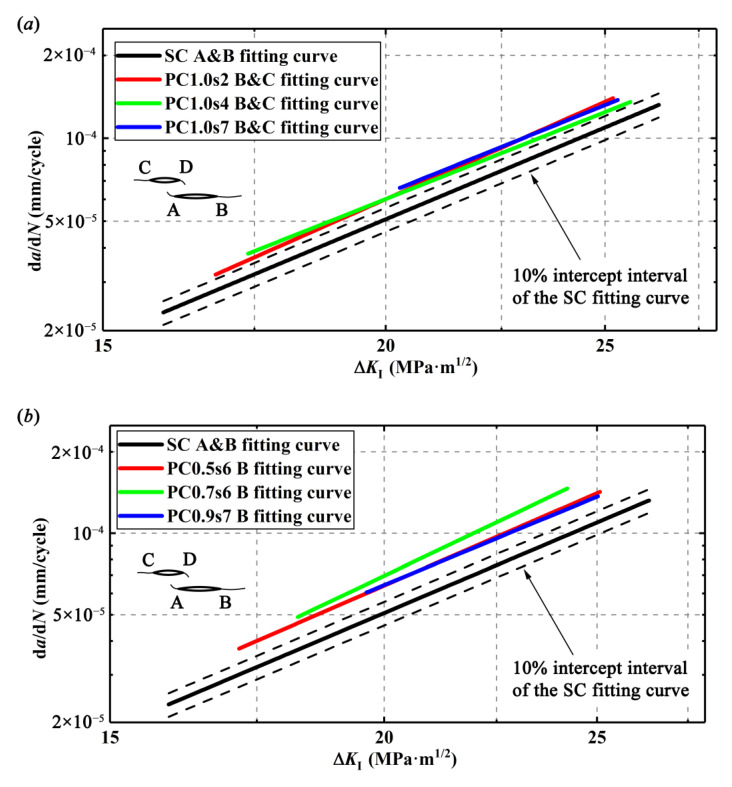

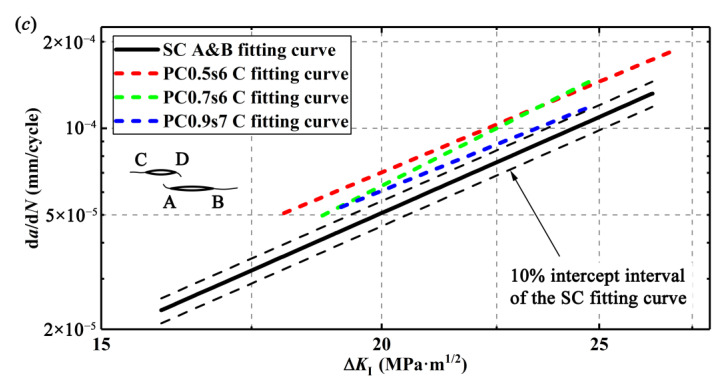
d*a*/d*N* vs. Δ*K*_I_: (**a**) for single crack and double equal cracks, (**b**) for single crack and crack tip B in double unequal cracks, and (**c**) for single crack and crack tip C in double unequal cracks.

The relationships between the driving force, Δ*K*_I_, which is calculated by EFM, and the crack growth rates, d*a*/d*N*, are linearly fitted from the experimental data in the double logarithm coordinate and thus, the Paris equations for the single crack and different double parallel cracks are obtained and compared as shown in Appendix A. All the fitted curves are plotted in Figure 11. Clearly, as a result of enhancement effect, for a given Δ*K*_I_, the outer crack tips of the double parallel cracks have higher CGRs than the single crack. It is usually considered that the parameters *c* and *m* in Paris equation are affected by the materials, but it turns out that they can also be affected by the crack configurations. However, only a few relevant studies can be found. For example, some researchers [28,29] also reported similar results that the double cracks have different Paris equations from the single crack.

## 5. Simplification of Double Cracks

**Figure 12:**
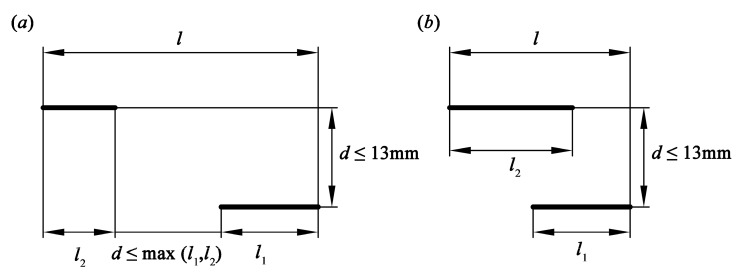
Method for simplification of double parallel cracks proposed by ASME: (**a**) nonoverlapping cracks and (**b**) overlapping cracks.

Double parallel cracks are usually simplified to a single crack in engineering applications, determined by the relative positions of the cracks. As shown in Figure 2 and Figure 3, the double parallel cracks finally propagate like a single crack as the inner crack tips propagate at much low rates. Regarding crack configuration simplification, there are several approaches in engineering, and the most typical one is proposed by ASME Boiler and Pressure Vessel Code Section XI [35]. It is found from Figure 12 that according to the ASME, if the normal distance of the parallel cracks is no more than 13 mm and the offset distance of the two inner crack tips is no more than the length of the long crack or the cracks are overlapped, the double parallel cracks of length *l*_1_ and *l*_2_ can be equivalent to a single crack of length *l*. Then, Paris equation of the single crack can be adopted to evaluate the problem of double parallel cracks.

According to the ASME method, the double parallel cracks can be equivalent to a single crack with the projected length perpendicular to the loading direction. For obtaining the driving force Δ*K*_I_* and crack growth rates d*a*/d*N* of the length-equivalent single crack, it is assumed that for the double equal crack specimens, crack growth lengths at the two crack tips of the length-equivalent single crack are separately equal to those at crack tips B and C. For the double unequal cracks, crack growth lengths at the two crack tips of the length-equivalent single crack are both equal to that of crack tip B, which will yield a conservative result because crack tip B grows faster than the other crack tip.

**Figure 13:**
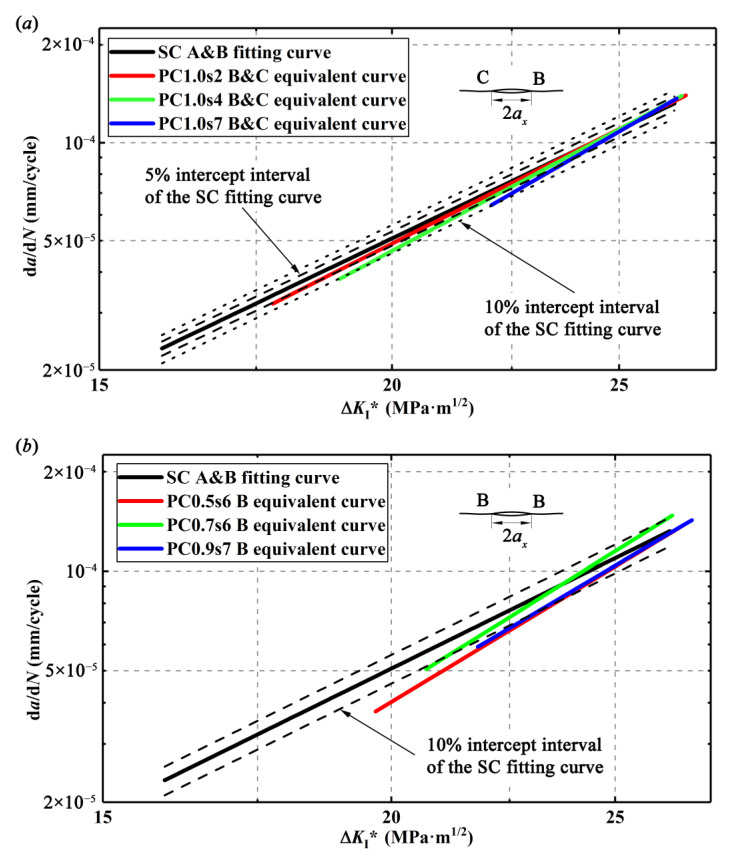
d*a*/d*N* vs. Δ*K*_I_^*^ for different specimens: (**a**) single crack and length-equivalent single crack from double equal cracks and (**b**) single crack and length-equivalent single crack from double unequal cracks.

With d*a*/d*N* and Δ*K*_I_*, d*a*/d*N*-Δ*K*_I_* curves for the length-equivalent single crack from the double parallel cracks with different specimens can be plotted as the so-called equivalent curves in Figure 13. It is found that the d*a*/d*N*-Δ*K*_I_* curves of the double parallel cracks are below the d*a*/d*N*-Δ*K*_I_* curve of the single crack (SC fitting curve in Figure 11) in Figure 13. For the double equal crack specimens, the differences of these two types of curves are within 10% but beyond 5% intercept interval, and for the double unequal crack specimens, the differences of these two types of curves are even beyond 10% intercept interval, implying that the ASME method is not so accurate. It seems that it is not enough to only modify the crack length regarding CGRs but the material parameters *c* and *m* in the Paris equation of the single crack should also be modified to obtain the CGRs of the double parallel cracks. Actually, it is understandable that the enhancement effect on the cracks may not only enhance the crack tip stress field or the SIFs at the tip but also increase the material damage around the crack tip and thus, affects the material parameters *c* and *m* in the Paris equation. In addition, it is found from Figure 13 that with the increase of Δ*K*_I_*, the difference of the CGRs between the single crack and the length-equivalent single crack from double parallel cracks gets smaller, implying that the growth behaviors of the double parallel cracks are gradually consistent with those of the single crack.

**Figure 14:**
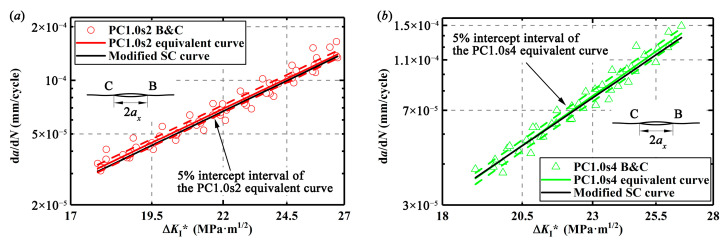

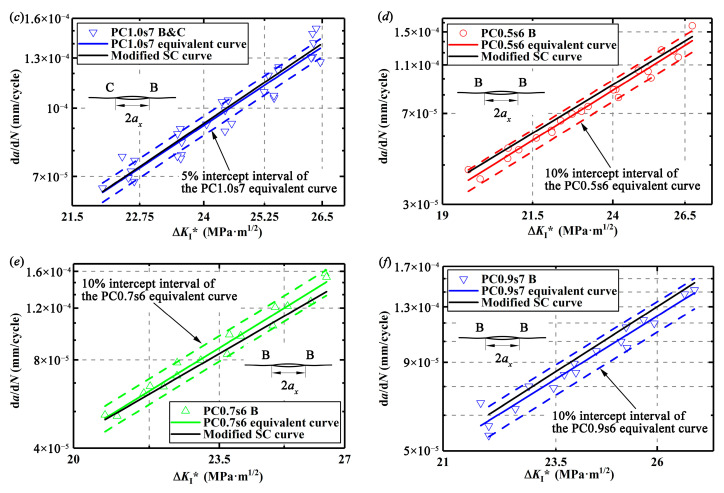
Comparison of simplification method and the ASME method for the double parallel cracks: (**a**) in PC1.0s2 specimens, (**b**) in PC1.0s4 specimens, (**c**) in PC1.0s7 specimens, (**d**) in PC0.5s6 specimens, (**e**) in PC0.7s6 specimens, and (**f**) in PC0.9s7 specimens.

The parameters *c* and *m* in the Paris equation of the single crack specimen and the parameters *c*’ and *m*’ in the Paris equations of the length-equivalent single crack from the double parallel crack specimens are obtained based on the fitting curves in Figure 14, as listed in Table 5 and Table 6.

With the least square method, the parameters *c* and *m* can be modified into *c*’ and *m*’ according to Equations (17)–(19), where *K*_I_ is the SIF at crack tip B of the double parallel cracks with the initial sizes and positions listed in Table 3 and *K*_I_’ is the SIF at the crack tips of the initial projected crack with the length of 2*a_x_*. In Equation (19), *K*_I_/*K*_I_^0^ can be obtained by Equations (14) and (15), where *a*_1_ is the initial half length of crack 1 and *a_x_* is the half length of the initial projected crack.

(17) $$m\prime =m/K_{R}{}^{0.722}$$

(18) $$\mathrm{lg}\left(c\prime \right)=\mathrm{lg}\left(c\right)/K_{R}{}^{0.414}$$

(19) $$K_{R}=\frac{K_{I}}{K_{I}’}=\frac{K_{I}}{K_{I}^{0}}\sqrt{\frac{a_{1}}{a_{x}}}$$

According to the modified material parameters *c*’ and *m*’ by Equations (17) and (18), d*a*/d*N*-Δ*K*_I_* curves based on the modified Paris equation are plotted and called the modified SC curve in Figure 14. The equivalent curves as shown in Figure 13 are also plotted in Figure 14. Obviously, the modified d*a*/d*N*-Δ*K*_I_* curves obtained from the simplification method proposed here are within 5% intercept interval of the equivalent curves for the double equal cracks as seen in Figure 14a–c and within 10% intercept interval for the double unequal cracks as seen in Figure 14d–f.

As mentioned above, the ASME method is to simplify the double parallel cracks into a single crack with an equivalent crack length. However, the simplification method proposed here is not only equalizing the crack length but also modifying the material parameters *c* and *m* in the Paris equation. Comparison results of the two methods are listed in Table 7, where *N*1 is the number of cycles from the fatigue crack propagation tests when crack tip B grows to a given length and *N*2 and *N*3 are the number of cycles obtained from the ASME method and the simplification method, respectively. Obviously, compared with the ASME method, the simplification method here is more accurate.

## 6. Conclusions

By performing the experiments and numerical simulations, interactions of double cracks are investigated in this study. Conclusions are obtained as follows:

1. Determined by different sizes and positions of the double cracks, interactions of the double parallel cracks present a shielding or enhancement effect on the SIFs and the CGRs at crack tips. For the double offset cracks, owing to the increase of the effective crack length, crack interactions mostly present enhancement effects.
2. According to the numerical simulation results, empirical formulas are obtained to calculate the SIF at the “dangerous” crack tip of the double parallel cracks by modifying the SIF at a single crack tip.
3. Crack enhancement interactions not only enhance the stress field but also increase the material damage around the crack tip and thus, affect the material parameters *c* and *m* in the Paris equation.
4. By modifying the material parameters *c* and *m* in Paris equation for the single crack, the double parallel cracks are simplified into a single crack with the same CGRs. This simplification method is more accurate compared with the ASME method.

## Figures and Tables

**Table 1:** Chemical composition of S30408 (wt%) [31].

Material	C	Si	Mn	P	S	Cr	Ni	N
S30408	≤0.08	≤0.75	≤2.00	≤0.035	≤0.015	18.0–20.0	8.0–10.5	0.10

**Table 2:** Mechanical properties of S30408 at the room temperature.

Young’s Modulus	Poisson’s Ratio	Proof Strength, Plastic Extension R_p0.2_	Tensile Strength	Percentage Elongation after Fracture
195 GPa ^1^	0.3 ^2^	≥220 MPa ^3^	≥520 MPa ^3^	≥40% ^3^

^1^ From reference [32], ^2^ from reference [33], and ^3^ from reference [31].

**Table 3:** Initial sizes and positions of the cracks in seven groups of specimens.

Specimens	SC	PC1.0s2	PC1.0s4	PC1.0s7	PC0.5s6	PC0.7s6	PC0.9s7
*a*_1_ (mm)	3	3	3	3	3	3	3
*a*_2_ (mm)	–	3	3	3	1.5	2.1	2.7
*s* (mm)	–	2	4	7	6	6	7
*h* (mm)	–	2.5	2.5	2.5	2.5	2.5	2.5

**Table 4:** Comparison results of the stress intensity factor (SIF) at crack tip B.

*Ra*	*S*	*H*	Values of *K*_I_ by Equations (14) and (15) (MPa·m^1/2^)	Values of *K*_I_ by FEM (MPa·m^1/2^)	Error (%)
0.5	0.467	0.1	12.673	12.184	4.01
0.7	1.567	0.1	14.396	14.920	3.52
0.9	2.133	0.1	14.000	13.420	4.32
1.0	2.333	0.1	14.025	13.383	4.79

**Table 5:** Material parameters in the Paris equations of the single crack specimen.

Parameters	*m*	*c*
SC	3.445	1.675 × 10^−9^

**Table 6:** Material parameters in the Paris equations of the length-equivalent single crack.

Specimens	PC1.0s2	PC1.0s4	PC1.0s7	PC0.5s6	PC0.7s6	PC0.9s7
*m*’	3.632	3.858	4.145	4.225	4.358	4.131
*c*’	9.229 × 10^−10^	4.445 × 10^−10^	1.736 × 10^−10^	1.282 × 10^−10^	9.283 × 10^−11^	1.744 × 10^−10^
*K_R_*	0.916	0.896	0.754	0.783	0.786	0.748

**Table 7:** Comparison of the two methods.

Specimens	Crack Growth Length (mm)	Test Results	The ASME Method		The Simplification Method	
		*N*1	*N*2	Error (%)	*N*3	Error (%)
PC1.0s2	8	178,449	169,243	5.16	185,351	3.87
PC1.0s4	7	128,922	117,809	8.62	128,742	0.14
PC1.0s7	6	79,000	71,987	8.88	78,622	0.48
PC0.5s6	7	136,257	109,216	19.85	126,812	6.93
PC0.7s6	7	112,451	99,936	11.13	113,802	1.20
PC0.9s7	6	86,258	75,053	12.99	82,763	4.05

**Figure A1:**
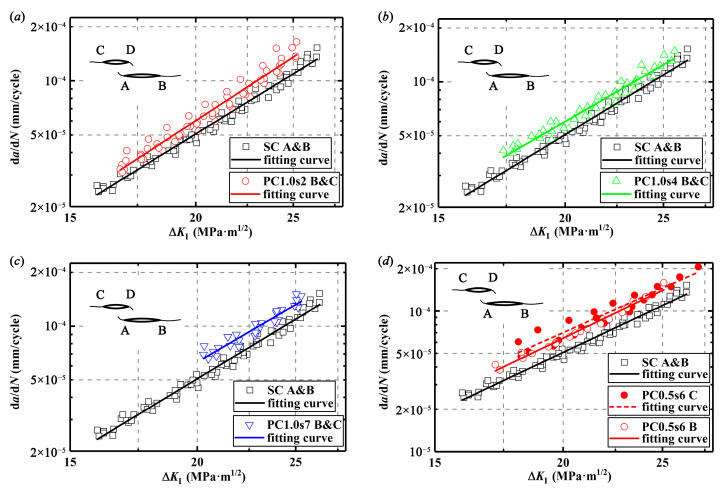

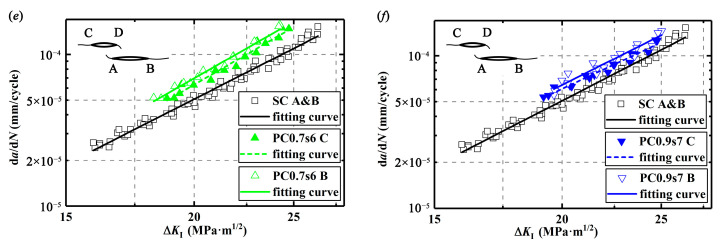
d*a*/d*N* vs Δ*K*_I_ for different specimens: (**a**) SC and PC1.0s2, (**b**) SC and PC1.0s4, (**c**) SC and PC1.0s7, (**d**) SC and PC0.5s6, (**e**) SC and PC0.7s6, and (**f**) SC and PC0.9s7.
